# Evaluating the Nephrotoxicity of Area-under-the-Curve-Based Dosing of Vancomycin with Concomitant Antipseudomonal Beta-Lactam Antibiotics: A Systematic Review and Meta-Analysis

**DOI:** 10.3390/medicina59040691

**Published:** 2023-03-31

**Authors:** Chia-Yu Chiu, Amara Sarwal

**Affiliations:** 1 Division of Infectious Diseases, Department of Internal Medicine, The University of Texas Health Science Center at Houston, Houston, TX 77030, USA; 2 Department of Infectious Diseases, Infection Control and Employee Health, The University of Texas MD Anderson Cancer Center, Houston, TX 77030, USA; 3 Division of Nephrology and Hypertension, University of Utah, Salt Lake City, UT 84132, USA; amara.sarwal@hsc.utah.edu

**Keywords:** vancomycin, piperacillin-tazobactam, area under the curve, vancomycin trough, acute kidney injury

## Abstract

*Background and Objectives*: Vancomycin combined with piperacillin/tazobactam (vancomycin + piperacillin/tazobactam) has a higher risk of acute kidney injury (AKI) than vancomycin combined with cefepime or meropenem. However, it is uncertain if applying area under the curve (AUC)-based vancomycin dosing has less nephrotoxicity than trough-based dosing in these combinations. *Materials and Methods*: We searched PubMed, Embase, Cochrane Library, and ClinicalTrials.gov from inception to December 2022. We examined the odds ratio (OR) of AKI between vancomycin + piperacillin/tazobactam and the control group. The control group was defined as vancomycin combined with antipseudomonal beta-lactam antibiotics, except for piperacillin-tazobactam. *Results*: The OR for AKI is significantly higher in vancomycin + piperacillin/tazobactam compared with the control group (3 studies, 866 patients, OR of 3.861, 95% confidence interval of 2.165 to 6.887, *p* < 0.05). In the sample population of patients who received vancomycin + piperacillin/tazobactam (2 studies, 536 patients), the risk of AKI (OR of 0.715, 95% CI of 0.439 to 1.163, *p* = 0.177) and daily vancomycin dose (standard mean difference—0.139, 95% CI—0.458 to 0.179; *p* = 0.392) are lower by AUC-based dosing than trough-based dosing, although it is not statistically significant. *Conclusions*: Nephrotoxicity is higher when combined with piperacillin/tazobactam than other antipseudomonal beta-lactam antibiotics (cefepime or meropenem) using the AUC-based dosing. However, applying the AUC-based dosing did not eliminate the risk of AKI or significantly reduce thedaily vancomycin dose compared with the trough-based dosing in the available literature.

## 1. Introduction

Vancomycin is a tricyclic glycopeptide antibiotic that targets methicillin-resistant *Staphylococcus aureus* (MRSA) [1]. Acute kidney injury (AKI) is a known major adverse effect of vancomycin [2]. This is even higher when it is concurrently administered with other nephrotoxic medications [3]. In patients with sepsis or nosocomial infection, vancomycin combined with various antibiotics is prescribed empirically to achieve broad-spectrum antimicrobial coverage, especially to cover *Pseudomonas aeruginosa* [3]. Vancomycin concomitantly administered with piperacillin/tazobactam is associated with a higher AKI rate than other combinations of vancomycin with antipseudomonal beta-lactam antibiotics [3,4].

Vancomycin therapeutic drug monitoring is a valuable strategy to achieve therapeutic efficacy and reduce the risk of vancomycin-induced nephrotoxicity. Compared with the 2009 Infectious Diseases Society of America (IDSA) consensus guideline, the latest 2020 IDSA consensus guideline supports area under the curve (AUC)-based vancomycin dosing (with a target of 400–600 mg h/L) more than trough-based dosing (with a target of 15–20 mg/L) [5,6]. AUC-based dosing provides a better approach to predicting and managing vancomycin than trough-based dosing [7]. Previous meta-analyses have shown that the AUC-based dosing method has a lower incidence of AKI than the trough-based dosing method [8]. However, the studies supporting this recommendation did not analyze the data from patients who received vancomycin + piperacillin/tazobactam [8,9]. The real-world data of AUC-based dosing when vancomycin is combined with antipseudomonal beta-lactam is largely unknown.

This meta-analysis aims to evaluate all the available literature on AUC-based vancomycin dosing combined with antipseudomonal beta-lactam antibiotics and the occurrence of AKI.

## 2. Methods

### 2.1. General Guidelines

We followed the guidelines delineated in the latest version of the PRISMA 2020 guidelines for this meta-analysis [10]. This study was registered in https://inplasy.com/inplasy-2022-12-0025/ (accessed on 6 December 2022, registered number: INPLASY2022120025).

### 2.2. Database Searches and Identification of Eligible Papers

Authors made independent electronic searches in PubMed, Embase, Cochrane Library, and ClinicalTrials.gov (accessed on 21 December 2022), which were searched from inception to 21 December 2022, using the following search protocol: ((Vancomycin area under the concentration-time curve) OR (Vancomycin AUC)) AND ((piperacillin-tazobactam) OR (ceftazidime) OR (cefoperazone-sulbactam) OR (cefepime) OR (imipenem-cilastatin) OR (doripenem) OR (meropenem) OR (beta-lactam)). The search strategy was identically applied to all databases. No language restrictions were imposed. The detailed search strategy for this systematic review and meta-analysis is provided in the Supplementary Materials (Supplementary Tables S1–S3).

### 2.3. Inclusion and Exclusion Criteria

Clinical studies reporting AKI in patients receiving vancomycin + piperacillin/tazobactam and vancomycin with another antipseudomonal beta-lactam were screened. All study types, except case reports, case series, and conference abstracts, were considered.

The exclusion criteria for this review and meta-analysis were as follows: (1) animal studies, (2) studies not designed to compare the vancomycin dosing method (AUC-based dosing vs. trough-based dosing), (3) studies not designed to compare vancomycin + piperacillin/tazobactam and a control group, or (4) review articles.

### 2.4. Methodological Quality Appraisal

The methodological quality of the enrolled studies was evaluated using the Newcastle–Ottawa Quality Assessment Scale (NOS). The NOS contains nine items in three categories: participant selection, comparability, and exposure. Studies with NOS scores ≥ 7 were considered high-quality studies; otherwise, they were reported as low-quality studies [11].

### 2.5. Data Extraction and Management

All eligible articles were reviewed. Authors extracted data from the recruited studies. First, the author, year, sample size, number and type of treatment arms, and participant characteristics were recorded. Data for the incidence of AKI and antimicrobial regimens were extracted from the published article or provided by authors upon request. Definitions for AKI varied in each article. In situations where the data were unavailable in the published article, we contacted the corresponding authors to request the original data.

Antipseudomonal beta-lactam antibiotics were defined as piperacillin/tazobactam, ceftazidime, cefoperazone-sulbactam, cefepime, imipenem-cilastatin, doripenem, and meropenem. The control group was defined as vancomycin combined with the above antipseudomonal beta-lactam antibiotics, except for piperacillin/tazobactam.

### 2.6. Primary and Secondary Outcomes

**Figure 1:**
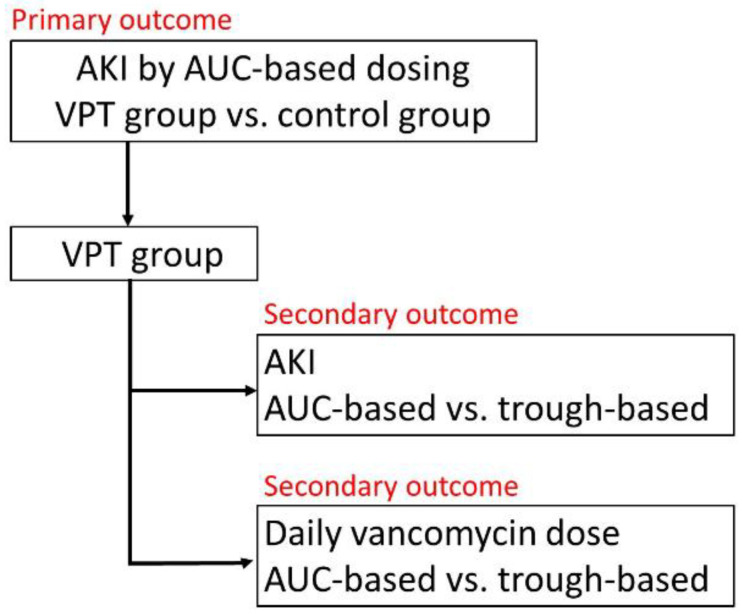
Scheme of this study design. AKI, acute kidney injury; AUC, area under the curve; VPT, vancomycin combined with piperacillin/tazobactam.

The primary outcome is the odds ratio (OR) of AKI in patients who received vancomycin + piperacillin/tazobactam or the control using the AUC-based vancomycin dosing. The secondary outcomes are (1) the OR of AKI in patients who received vancomycin + piperacillin/tazobactam using AUC-based vancomycin dosing or trough-based dosing (reference method) and (2) the daily vancomycin dose in patients who received vancomycin + piperacillin/tazobactam using AUC-based dosing or trough-based dosing (reference method). The scheme of this study design is shown in Figure 1.

### 2.7. Statistical Analysis

Based on the heterogeneous target populations in the recruited studies, the meta-analysis was conducted using a random-effects model [12]. Between-trial heterogeneity was determined using I^2^ tests; an I^2^ > 50% was considered statistically significant heterogeneity. Funnel plots and the Egger’s test were used to examine the potential publication bias. The potential publication bias was evaluated according to the *Cochrane Handbook for Systematic Reviews of Interventions* [13]. We visually inspected funnel plots when there were fewer than 10 datasets. Egger’s regression tests were performed when there were 10 or more datasets. Statistical significance was defined as *p* values < 0.05, except for the determination of publication bias, which employed *p* < 0.10. All analyses were performed using comprehensive meta-analysis (CMA) software, version 3.3 (Biostat, Englewood, NJ, USA).

## 3. Results

### 3.1. Study Selection

**Figure 2:**
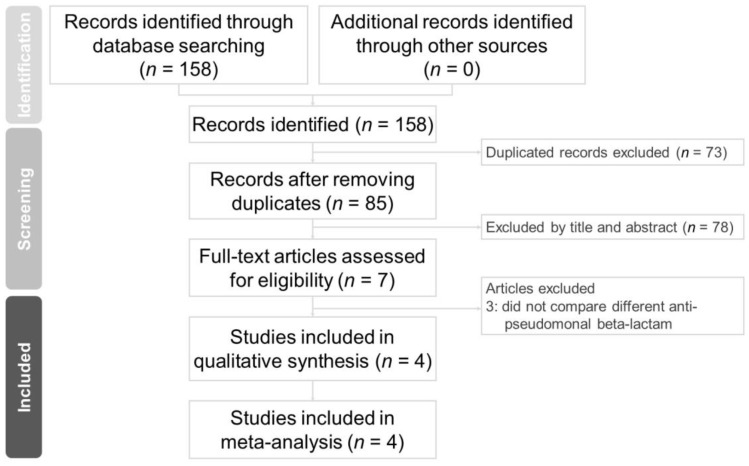
PRISMA 2020 flowchart of the current meta-analysis.

We retrieved 85 non-duplicated citations for a review of their titles and abstracts. Based on our included criteria, four articles were selected for meticulous evaluation. The PRISMA flowchart of the literature search process is presented in Figure 2. Characteristics of the studies included are summarized in Table 1. All four studies are retrospective and focused on the adult population (age > 18 years old). Among these four studies, one is from Japan, and the remaining three are from the United States.

### 3.2. Methodological Quality of the Included Studies

Three studies had an NOS score of 6 and one study had an NOS score of 7. The main risk bias came from (1) a lack of AUC-level reports and (2) the fact that the control group did not separate different antipseudomonal beta-lactam antibiotics. The detailed Newcastle–Ottawa Quality Assessment Scale is summarized in the Supplementary Materials (Supplementary Table S4).

### 3.3. Primary Outcome: Risk of AKI in Patients Who Received Vancomycin + Piperacillin/Tazobactam under AUC-Based Dosing

**Figure 3:**
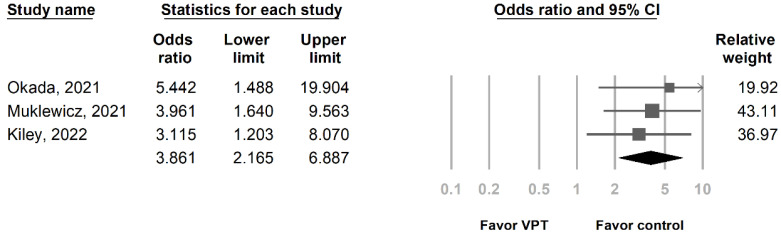
Forest plot presenting the OR of AKI by AUC-based dosing in patients who received VPT and control antibiotic. AKI, acute kidney injury; AUC, area under the curve; OR, odds ratio; VPT, vancomycin combined with piperacillin/tazobactam.

In total, 3 studies included 866 patients who were evaluated for AKI (Table 1) [14,15,16]. Patients were separated into the vancomycin + piperacillin/tazobactam group (510 patients) and control group (356 patients). The control group included vancomycin combined with cefepime (vancomycin + cefepime) and vancomycin combined with meropenem (vancomycin + meropenem) (Table 1). The OR for AKI was significant in the vancomycin + piperacillin/tazobactam group compared with the control group (OR of 3.861, 95% confidence interval (CI) of 2.165 to 6.887, *p* < 0.05) (Figure 3), and the individual studies had low heterogeneity (I^2^ = 0%, *p* = 0.792). An Egger’s test was not performed because only three studies were included in this analysis.

### 3.4. Secondary Outcomes: Risk of AKI and Daily Vancomycin Dose in Patients Who Received Vancomycin + Piperacillin/Tazobactam under Different Vancomycin Dosing

**Figure 4:**
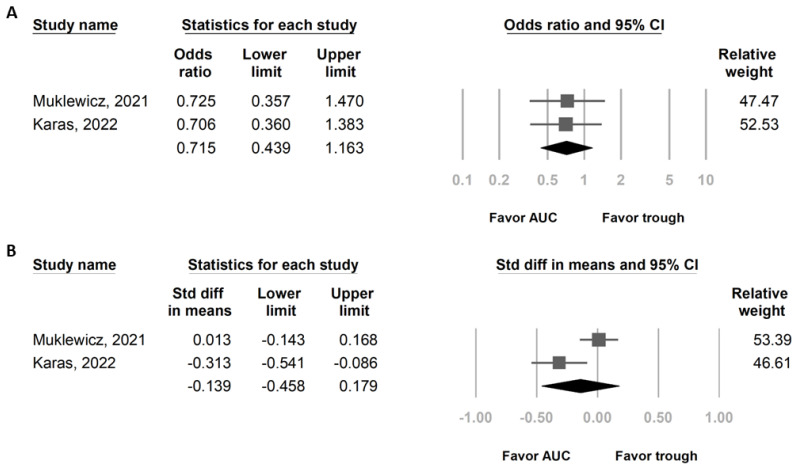
Forest plot presenting (**A**) OR of AKI and (**B**) total daily vancomycin dose in patients who received VPT by AUC-based dosing and trough-based dosing. AKI, acute kidney injury; AUC, area under the curve; OR, odds ratio; VPT, vancomycin combined with piperacillin/tazobactam.

In total, 2 studies that included a total of 536 patients who received vancomycin + piperacillin/tazobactam were evaluated (Table 1) [15,17]. Both articles provide different definitions of AKI in their outcome. We decided to use the IDSA definition [5]. AKI is defined by the IDSA as an increase in serum creatinine of 0.5 mg/dL or a 50% increase from baseline on two or more consecutive daily measurements [5]. Patients were separated into AUC-based dosing (268 patients) and trough-based dosing (268 patients). Although not statistically significant, the OR for AKI was lower in AUC-based dosing than in trough-based dosing (OR of 0.715, 95% CI of 0.439 to 1.163, *p* = 0.177) (Figure 4A). The total daily dose of vancomycin was lower in AUC-based dosing than in trough-based dosing, but there was no statistically significant difference (standard mean difference—0.139, 95% CI—0.458 to 0.179, *p* = 0.392) (Figure 4B). Heterogeneity and Egger’s tests were not performed because only two studies were included in this analysis.

## 4. Discussion

To our knowledge, this is the first meta-analysis that evaluated AUC-based dosing in patients who received vancomycin + piperacillin/tazobactam or vancomycin concurrent with other antipseudomonal beta-lactam antibiotics. We found that VPT still has a higher risk of AKI than vancomycin combined with other antipseudomonal beta-lactam antibiotics (cefepime and meropenem) by AUC-based dosing. In patients who received vancomycin + piperacillin/tazobactam, the risk of AKI and daily vancomycin dose is lower within this sample population by AUC-based dosing than trough-based dosing, although not statistically significant.

The four retrospective studies included in our analysis were published in 2021 and 2022, reflective of the recent change in vancomycin dosing with the AUC-based method being advocated in the 2020 IDSA guideline [6]. Three studies were analyzed for the primary outcome and showed a significantly higher risk of AKI with vancomycin + piperacillin/tazobactam than the control group (Figure 3). We could not perform the subgroup analysis for vancomycin + cefepime and vancomycin + meropenem individually due to control groups receiving both vancomycin + cefepime and vancomycin + meropenem in two of the three studies [15,16]. This was due to providers escalating antibiotics from vancomycin + cefepime to vancomycin + meropenem or de-escalating antibiotics from vancomycin + meropenem to vancomycin + cefepime.

Two retrospective studies were analyzed for the secondary outcome, revealing that AUC-based dosing had a numerically lower risk of AKI (both studies used the IDSA definition of AKI) than trough-based dosing (Figure 4A). A prospective observational study, which included 117 patients on AUC-based vancomycin dosing, revealed a higher risk of developing AKI when exposed to piperacillin/tazobactam [18]. Therefore, when evaluating vancomycin-indued nephrotoxicity in AUC-based dosing, the consideration of concomitant drugs as confounders is important. A higher vancomycin daily dose is associated with a higher risk of AKI, and patients with trough-based dosing received a higher daily dose of vancomycin than AUC-based dosing [9,18,19]. This trend was also found in our analysis, although no statistically significant difference was found (Figure 4B). Perhaps AUC-based dosing can decrease the amount of daily vancomycin used and overcome the nephrotoxicity of piperacillin/tazobactam.

Although the 2020 IDSA guideline suggests AUC-based vancomycin dosing with a target of 400–600 mg h/L for MRSA minimum inhibitory concentration ≤1, one prospective observational study showed significant nephrotoxicity when AUC level ≥550 (mg h/L) in the treatment of MRSA bacteremia [20]. It is unknown if the AUC level cutoff of 550 (mg h/L) versus 600 (mg h/L) can be applied in empiric broad-spectrum antibiotic coverage (i.e., vancomycin + piperacillin/tazobactam, vancomycin + cefepime, or vancomycin + meropenem) in patients with sepsis, nosocomial infection, osteomyelitis, and intra-abdominal infection [3,15,21,22]. Perhaps the AUC-based dosing method could become a modifiable AKI risk factor in patients who received vancomycin + piperacillin/tazobactam.

The association of AKI and vancomycin + piperacillin/tazobactam was derived from the literature before the introduction and mainstream application of AUC-based dosing [4,23]. With the increasing use and validity of this novel method, clinicians should revisit the possibility of vancomycin + piperacillin/tazobactam administration to expand our armamentarium for broad-spectrum empiric therapy. There are two major knowledge gaps regarding area under the curve -based dosing of vancomycin in combination with antipseudomonal beta-lactam antibiotics. First, when combining vancomycin with antipseudomonal beta-lactam antibiotics, it remains to be seen whether AUC-based dosing is better than trough-based dosing to avoid nephrotoxicity. More studies are needed to assess whether AUC-based dosing decreases daily vancomycin dose and if that contributes to a lower risk of AKI in patients who received vancomycin + piperacillin/tazobactam. Second, the optimal vancomycin AUC level associated with decreased nephrotoxicity when combining vancomycin with antipseudomonal beta-lactam antibiotics still needs to be elucidated. Prospective or quasi-experimental studies are needed to assess other clinical outcomes (for example, length of stay or mortality) in the AUC-based dosing of vancomycin combined with antipseudomonal beta-lactam antibiotics. In addition, studies are needed to ascertain the cost-effectiveness of AUC-based vancomycin dosing when combined with antipseudomonal beta-lactam antibiotics.

There are several limitations of this meta-analysis. First, all the analyzed studies are retrospective with a relatively small sample size and were published in the past two years, after the IDSA 2020 recommendation. Second, there were inconsistencies with the definition of AKI utilized in each study. Ideally, the use of the same definition of AKI among the studies can eliminate the reporting bias. However, the different definitions of AKI might not change our meta-analysis results. Two studies included in our analysis compared the outcome utilizing different definitions of AKI but yielded the same results [15,17]. Third, different AUC calculation models have been used in these studies, even though the Bayesian model is the most commonly used. There are many AUC-based dosing tools, including some commercial software embedded in electronic medical record systems. Although the latest IDSA guidelines suggested Bayesian-derived AUC monitoring, it remains controversial about which equation has better efficacy and cost-effectiveness [6,24,25]. Fourth, not all antipseudomonal beta-lactam antibiotics are examined in this review because only cefepime and meropenem are found in the literature. Fifth, all included studies used creatinine to estimate kidney function. Cystatin C-estimated kidney function suggests that the observed association between vancomycin + piperacillin/tazobactam and creatinine-defined acute kidney injury may be pseudotoxicity. [26]. Sixth, AUC-based dosing remains as clinically effective as trough-based dosing in general [6]. Adding piperacillin/tazobactam, an antipseudomonal antibiotic, should not theoretically affect clinical efficacy, except for the risk of AKI. However, during our search, there were no articles specifically focused on clinical efficacy in this field.

In conclusion, patients who received vancomycin + piperacillin/tazobactam with AUC-based dosing had a higher risk of AKI than vancomycin combined with other antipseudomonal beta-lactam antibiotics (cefepime and meropenem). The application of the AUC-based vancomycin dosing did not eliminate the risk of AKI compared with the trough-based dosing in the available literature.

## Figures and Tables

**Table 1:** Characteristics of the four articles included in this study. AKI, acute kidney injury; AKIN: acute kidney injury network; AUC, area under curve (mg h/L); C, control group; IDSA: Infectious Disease Society of America; KDIGO, Kidney Disease: Improving Global Outcomes; NA, not available; RIFLE, risk, injury, failure, loss, end-stage kidney disease; VPT, vancomycin + piperacillin/tazobactam.

Author, Year, Country, Reference	Okada, 2021, Japan, [14]	Muklewicz, 2021, USA, [15]	Kiley, 2022, USA, [16]	Karas, 2022, USA, [17]
**Study Design**	Retrospective, single hospital	Retrospective, 3 hospitals	Retrospective, single hospital	Retrospective, 3 hospitals
**Period**	04/2010–03/2020	case: 04/2019–07/2019; control: 08/2019–03/2020	10/2019–09/2020	01/2015–06/2021
**Sample size**	VPT: 61, C ^a^: 42	VPT: 118, C ^b^: 210; AUC-based: 118, trough-based: 118	VPT: 331, C ^c^: 104	AUC-based: 150, trough-based: 150
**AUC calculation method**	Bayesian model	Bayesian model	Bayesian model or Sawchuk–Zaske method	Two-sample AUC or Bayesian model
**Actual AUC level**	VPT: median 452; C ^a^: median 427.7	AUC mean 445.7	VPT: mean 503; C ^c^: mean 495,	NA
**Duration of combination therapy**	VPT: median 5 days, C ^a^: median 4 days	VPT: mean 6.2 days, C ^b^: mean 4.8 days; AUC-based: mean 4.0 days, trough-based: mean 4.9 days	VPT: median 4 days, C ^c^: median 4 days	AUC-based: mean 5.18 days; trough-based: mean 5.11 days
**AKI definition**	KDIGO	IDSA/AKIN/RIFLE	RIFLE	IDSA/KDIGO/RIFLE
**Other outcomes examined**	AUC cutoff: 600	30 days readmission, in-hospital mortality	AUC cutoff: 550	No

^a^ Control group included vancomycin + cefepime. ^b^ Control group included vancomycin alone, vancomycin + cefepime, or vancomycin + meropenem. The control group regimens could not be analyzed as subgroups because they involve antibiotic escalation or de-escalation. This information is obtained directly from the reference article’s author and is not mentioned in the original manuscript. ^c^ Control groups included vancomycin + cefepime or vancomycin + meropenem. The control group regimens could not be analyzed as subgroups because they involve antibiotic escalation. This information is obtained directly from the reference article’s author and is not mentioned in the original manuscript.

## Data Availability

The datasets generated during and/or analyzed during the current study are available from the corresponding authors on reasonable request.

## Supplementary Materials

The following supporting information can be downloaded at: https://www.mdpi.com/article/10.3390/medicina59040691/s1, Table S1: PRISMA Checklist; Table S2. Keywords and search results in different databases; Table S3. Excluded studies and reasons; Table S4: Newcastle-Ottawa Quality Assessment Scale for included studies.
